# Supplementary material for: Knowledge and attitude towards mpox: Systematic review and meta-analysis
Source: PLoS One. 2024 Aug 9;19(8):e0308478. doi: 10.1371/journal.pone.0308478 (PMC11315308; doi:10.1371/journal.pone.0308478)
Supplement: S2 Table — (DOCX) [file pone.0308478.s002.docx]

**Table S2.** The adjusted search terms as per searched electronic databases.

| **PubMed** | | | |
| --- | --- | --- | --- |
|  | #1 | (Monkeypox OR “Monkey Pox”) | 3,412 |
|  | #2 | (“Knowledge”) | 1,005,183 |
|  | #3 | (“ Attitude” OR “ Attitudes” OR “Sentiment” OR “Sentiments” OR “Opinions” OR “Opinion”) | 608,550 |
|  | #4 | #1 AND #2 AND #3 | 48 |
| **Scopus** | | | |
|  | #1 | TITLE-ABS-KEY ( monkeypox OR "Monkey Pox" ) | 3,965 |
|  | #2 | TITLE-ABS-KEY ( "Knowledge" ) | 2,690,119 |
|  | #3 | TITLE-ABS-KEY ( " Attitude" OR " Attitudes" OR "Sentiment" OR "Sentiments" OR "Opinions" OR "Opinion" ) | 1,411,155 |
|  | #4 | #1 AND #2 AND #3 | 53 |
| **Embase** | | | |
|  | #1 | 'monkeypox'/exp OR 'monkeypox' | 4,147 |
|  | #2 | 'knowledge' | 1,219,506 |
|  | #3 | 'attitude' | 541,342 |
|  | #4 | #1 AND #2 AND #3 | 40 |
| **Web of Science** | | | |
|  | #1 | ALL=(Monkeypox OR “Monkey Pox”) | 3,450 |
|  | #2 | ALL= (“Knowledge”) | 2,001,028 |
|  | #3 | ALL= (“ Attitude” OR “ Attitudes” OR “Sentiment” OR “Sentiments” OR “Opinions” OR “Opinion”) | 707,095 |
|  | #4 | #1 AND #2 AND #3 | 54 |
| **ScienceDirect** | | | |
|  | #1 | (Monkeypox OR “Monkey Pox”) | 1,016 |
|  | #2 | (“Knowledge”) | 421,556 |
|  | #3 | (“ Attitude” ) | 78,759 |
|  | #4 | #1 AND #2 AND #3 | 104 |
