# Supplementary material for: Knowledge and attitude towards mpox: Systematic review and meta-analysis
Source: PLoS One. 2024 Aug 9;19(8):e0308478. doi: 10.1371/journal.pone.0308478 (PMC11315308; doi:10.1371/journal.pone.0308478)
Supplement: S3 Table — (DOCX) [file pone.0308478.s003.docx]

**Table S3**. Quality of the included studies

| Authors | Year | Eligibility criteria | Study subjects and the setting | Exposure measured in a valid and reliable way 'gold standard' | A specified diagnosis or definition | Confounding factors | Dealing with confounding factors | Outcomes measured in a valid and reliable way | Appropriate statistical analysis | Scores (8) | Quality (high, moderate, low) |  |
| --- | --- | --- | --- | --- | --- | --- | --- | --- | --- | --- | --- | --- |
| Hasan M, et al. [1] | | 2023 | Yes | Yes | Yes | Yes | Unclear | NA | Yes | Yes | 6 | Moderate |
| Swed S, et al. [2] | | 2023 | Yes | Yes | Yes | Yes | Unclear | NA | Yes | Yes | 6 | Moderate |
| Dong C, et al. [3] | | 2023 | Yes | Yes | Yes | Yes | Unclear | NA | Yes | Yes | 6 | Moderate |
| Swed S, et al. [4] | | 2023 | Yes | Yes | Yes | Yes | Unclear | NA | Yes | Yes | 6 | Moderate |
| Peng X, et al. [5] | | 2023 | Yes | Yes | Yes | Yes | Unclear | NA | Yes | Yes | 6 | Moderate |
| Berdida DJE, et al. [6] | | 2023 | Yes | Yes | Yes | Yes | Unclear | NA | Yes | Yes | 6 | Moderate |
| Elkhwesky Z, et al. [7] | | 2023 | Yes | Yes | Yes | Yes | Unclear | NA | Yes | Yes | 6 | Moderate |
| Youssef D, et al. [8] | | 2023 | Yes | Yes | Yes | Yes | Unclear | NA | Yes | Yes | 6 | Moderate |
| Malaeb D, et al. [9] | | 2023 | Yes | Yes | Yes | Yes | Unclear | NA | Yes | Yes | 6 | Moderate |
| Chen Y, et al. [10] | | 2023 | Yes | Yes | Yes | Yes | Unclear | NA | Yes | Yes | 6 | Moderate |
| Lounis M, et al. [11] | | 2023 | Yes | Yes | Yes | Yes | Unclear | NA | Yes | Yes | 6 | Moderate |
| Ahmed SK, et al. [12] | | 2023 | Yes | Yes | Yes | Yes | Unclear | NA | Yes | Yes | 6 | Moderate |
| Das SK, et al. [13] | | 2023 | Yes | Yes | Yes | Yes | Unclear | NA | Yes | Yes | 6 | Moderate |
| Al-Mustapha AI, et al. [14] | | 2023 | Yes | Yes | Yes | Yes | Unclear | NA | Yes | Yes | 6 | Moderate |
| Ren F, et al. [15] | | 2023 | Yes | Yes | Yes | Yes | Unclear | NA | Yes | Yes | 6 | Moderate |
| Alrasheedy AA, et al. [16] | | 2023 | Yes | Yes | Yes | Yes | Unclear | NA | Yes | Yes | 6 | Moderate |
| Sahin TK, et al. [17] | | 2022 | Yes | Yes | Yes | Yes | Unclear | NA | Yes | Yes | 6 | Moderate |
| Alshahrani NZ, et al. [18] | | 2022 | Yes | Yes | Yes | Yes | Unclear | NA | Yes | Yes | 6 | Moderate |
| Jairoun AA, et al. [19] | | 2022 | Yes | Yes | Yes | Yes | Unclear | NA | Yes | Yes | 6 | Moderate |
| Kaur A, et al. [20] | | 2022 | Yes | Yes | Yes | Yes | Unclear | NA | Yes | Yes | 6 | Moderate |
| Alshahrani NZ, et al. [21] | | 2022 | Yes | Yes | Yes | Yes | Unclear | NA | Yes | Yes | 6 | Moderate |
| Kumar N, et al. [22] | | 2022 | Yes | Yes | Yes | Yes | Unclear | NA | Yes | Yes | 6 | Moderate |
| Ajman F, et al. [23] | | 2022 | Yes | Yes | Yes | Yes | Unclear | NA | Yes | Yes | 6 | Moderate |
| Alshahrani NZ, et al. [24] | | 2022 | Yes | Yes | Yes | Yes | Unclear | NA | Yes | Yes | 6 | Moderate |
| Harapan H, et al. [25] | | 2020 | Yes | Yes | Yes | Yes | Unclear | NA | Yes | Yes | 6 | Moderate |
| Harapan H, et al. [26] | | 2020 | Yes | Yes | Yes | Yes | Unclear | NA | Yes | Yes | 6 | Moderate |
| Harapan H, et al. [27] | | 2020 | Yes | Yes | Yes | Yes | Unclear | NA | Yes | Yes | 6 | Moderate |

NA: Not assessed
