# Supplementary figures and images for: Knowledge and attitude towards mpox: Systematic review and meta-analysis
Source: PLoS One. 2024 Aug 9;19(8):e0308478. doi: 10.1371/journal.pone.0308478 (PMC11315308; doi:10.1371/journal.pone.0308478)

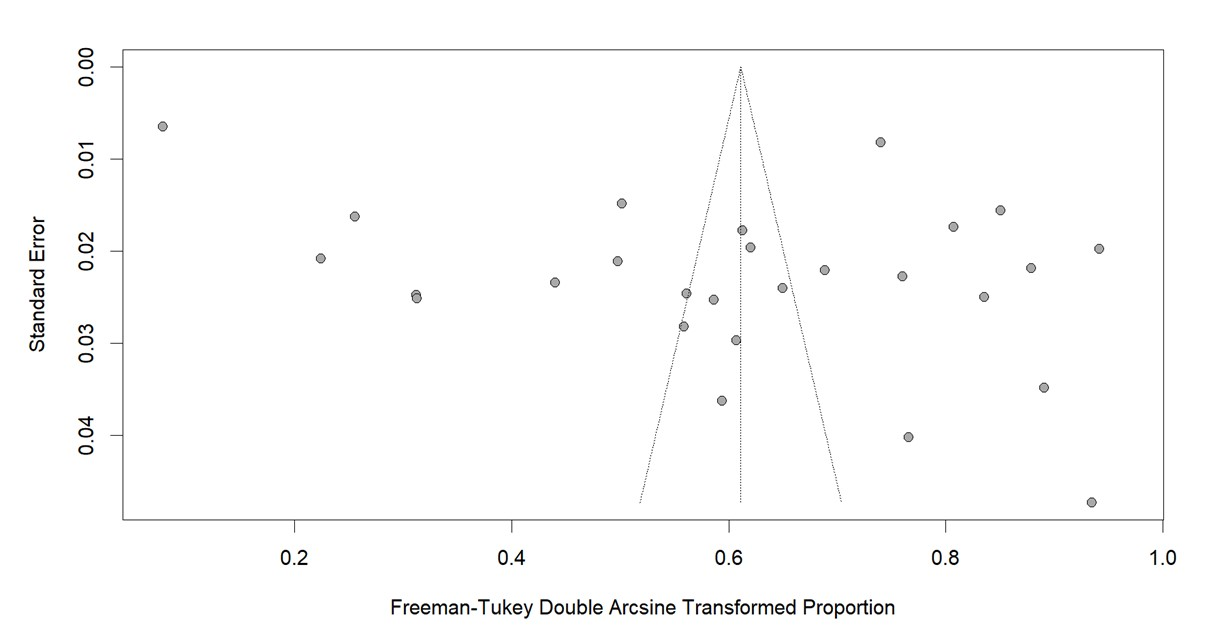

Supplement: S1 Fig — (TIF) [file pone.0308478.s004.tif]

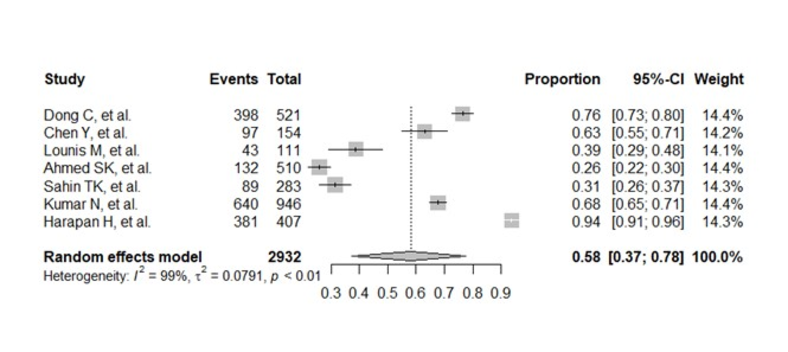

Supplement: S2 Fig — (TIF) [file pone.0308478.s005.tif]

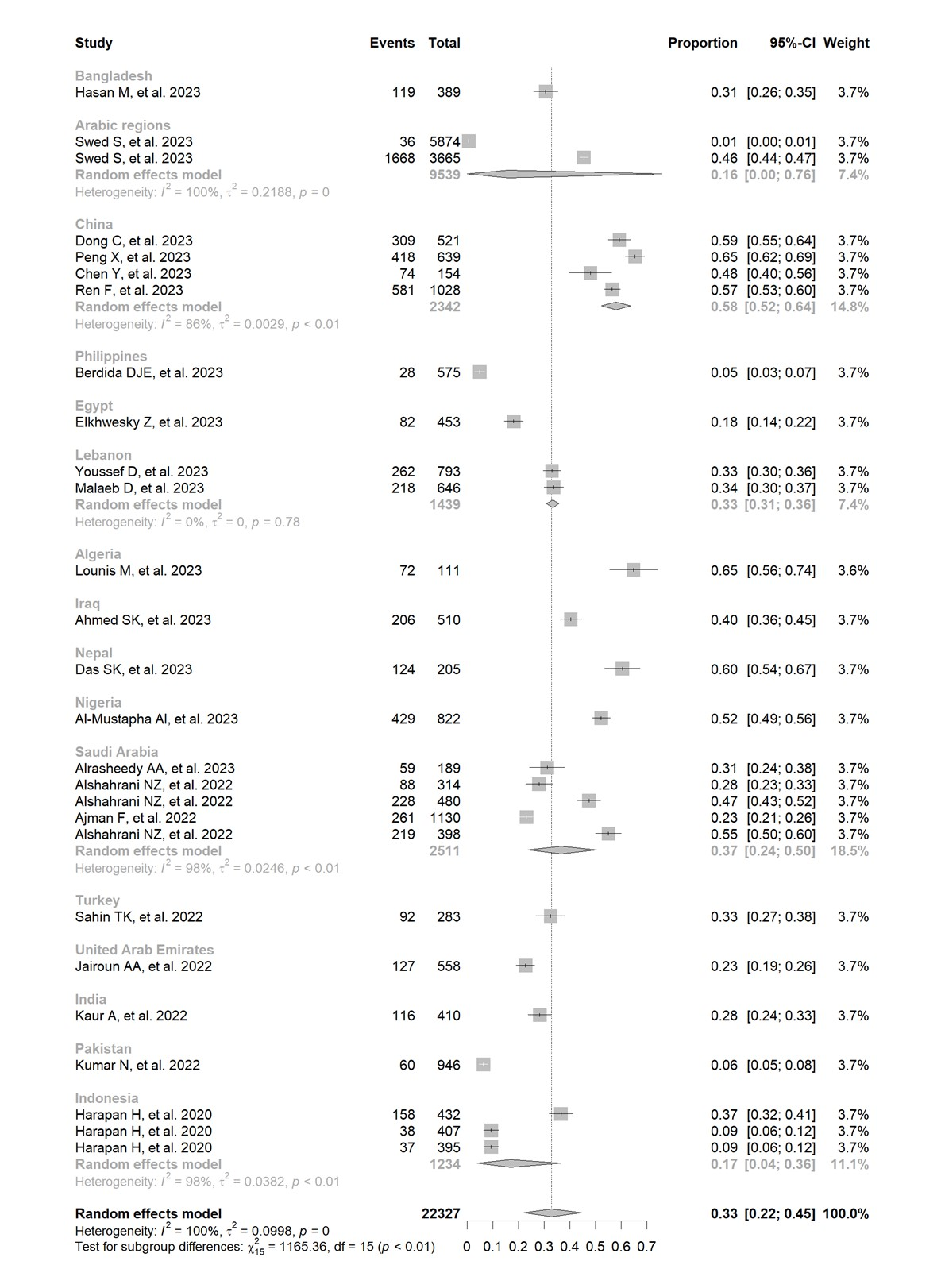

Supplement: S3 Fig — (TIF) [file pone.0308478.s006.tif]

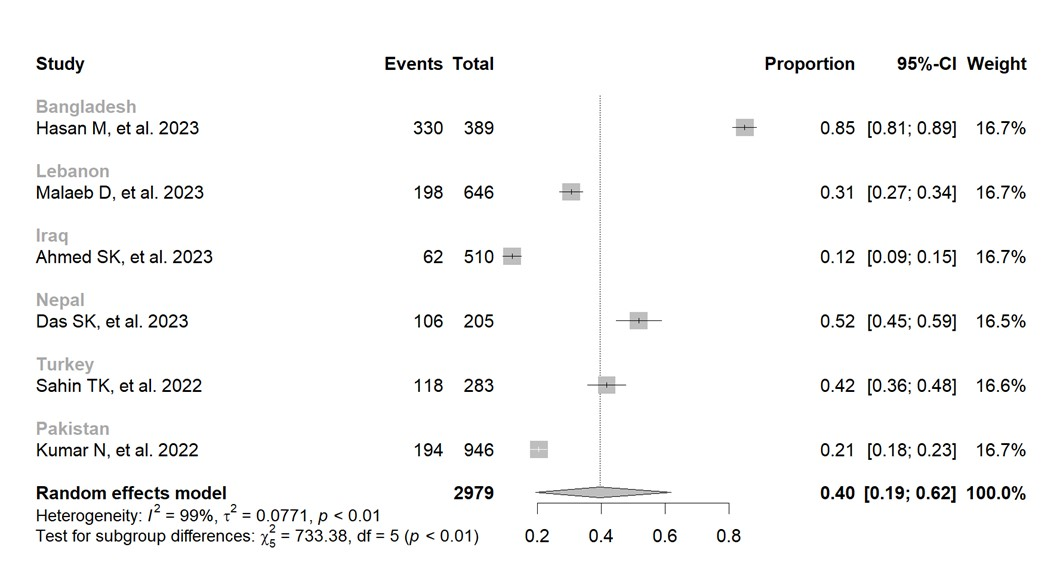

Supplement: S4 Fig — (TIF) [file pone.0308478.s007.tif]

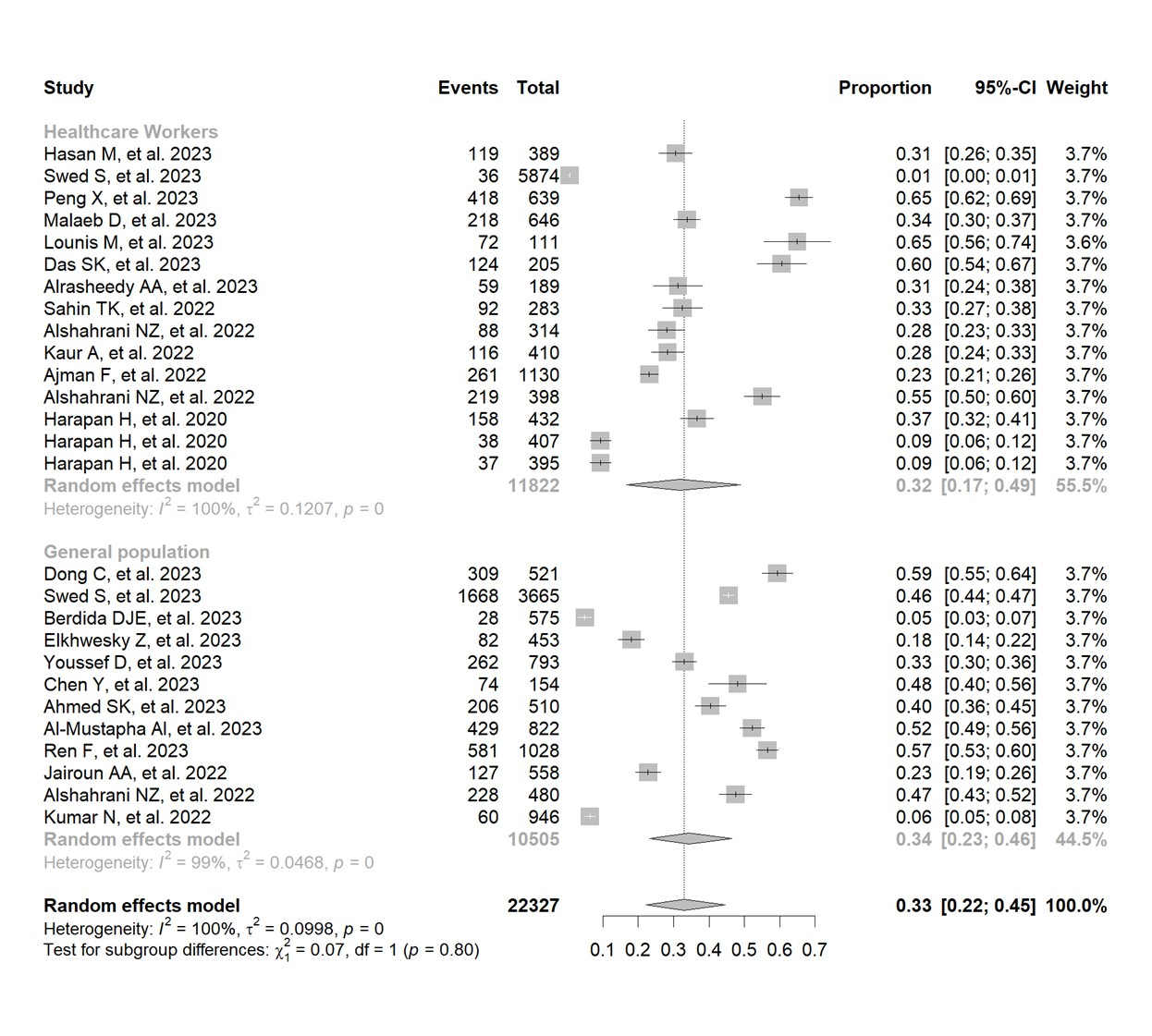

Supplement: S5 Fig — (TIF) [file pone.0308478.s008.tif]

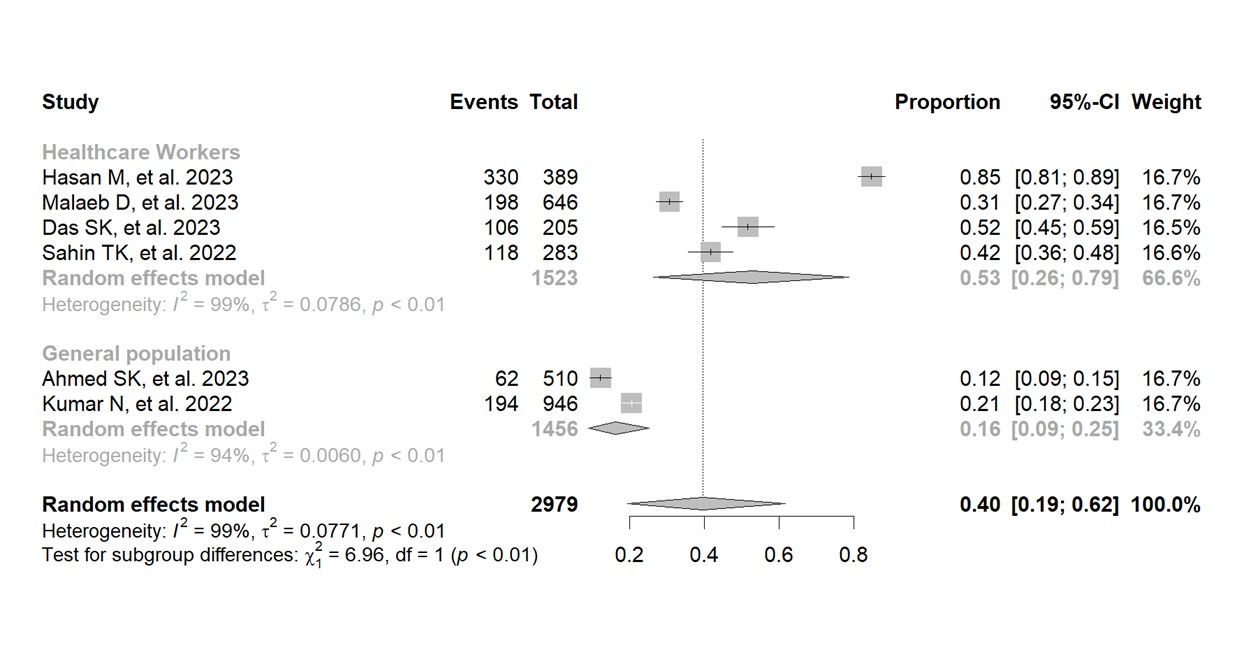

Supplement: S6 Fig — (TIF) [file pone.0308478.s009.tif]
